# Systemic Melioidosis with Acute Osteomyelitis and Septic Arthritis Misdiagnosed as Tuberculosis: A Case Report

**DOI:** 10.7759/cureus.7011

**Published:** 2020-02-16

**Authors:** Rohit Prasad, Nishan B Pokhrel, Suresh Uprety, Himal Kharel

**Affiliations:** 1 Orthopaedics, Tribhuvan University Institute of Medicine, Kathmandu, NPL; 2 Internal Medicine, Tribhuvan University Institute of Medicine, Kathmandu, NPL; 3 Clinical Pharmacology, Tribhuvan University Institute of Medicine, Kathmandu, NPL

**Keywords:** burkholderia pseudomallei, melioidosis, osteomyelitis, septic arthritis, tuberculosis

## Abstract

Melioidosis, also called Whitmore's disease, is an infectious disease caused by the bacterium Burkholderia pseudomallei. It is predominantly a disease of tropical climates, especially in Southeast Asia and northern Australia. Due to a wide range of signs and symptoms that can be mistaken for other diseases such as tuberculosis or common forms of pneumonia, patients can be frequently misdiagnosed, which can have adverse consequences and can make management more complicated. This case report elaborates on the clinical course of a middle-aged nondiabetic male patient who presented to our hospital with fever for two months and painful swelling of the right proximal leg for 10 days, following a previous diagnosis of disseminated abdominal tuberculosis made at a different healthcare center. Preliminary investigations confirmed multiple diagnoses of acute osteomyelitis and septic arthritis complicated by multiple hepatic and splenic abscesses. Given the patient was in a state of septic shock at the time of presentation, he was managed as an emergency case and an arthrotomy of the knee joint was performed followed by decompression and drainage of the right proximal tibia. As per standard hospital protocol, the pus and synovial fluid were sent for microbial culture and sensitivity, at which point B. pseudomallei was isolated and the diagnosis was confirmed. Diagnosis of melioidosis requires a high degree of suspicion among clinicians and microbiologists, especially in individuals that have frequent exposure to contaminated soil and water and have a travel history to endemic countries.

## Introduction

Melioidosis is an infectious disease of humans and animals caused by an environmental Gram-negative bacillus *Burkholderia pseudomallei* found in moist soil and water [1]. It is called 'the remarkable imitator' [2] due to its varied manifestations, ranging from localized skin abscess without systemic illness, to fulminant septicemia with abscess involving multiple internal organs and musculoskeletal system [3]. Often, it gets misdiagnosed and mistreated for tuberculosis in nonendemic areas [4]. Reports from a 20-year prospective study in Australia revealed a 50% case fatality rate in patients with septic shock and only 4% in the absence of septic shock [3]. Thus, the misdiagnosis of this disease has serious consequences.

Herein, we report a case of systemic melioidosis in a nondiabetic male, with travel history to Malaysia and South India, who presented with chronic fever and acute painful swelling of the right proximal leg. He was suspected to be suffering from disseminated abdominal tuberculosis in the previous center for which antitubercular drugs were initiated. We instituted appropriate therapy after the etiologic agent was identified during investigations. He responded well to treatment and did well during follow-up.

## Case presentation

A 38-year-old male was brought in our ED with complaints of fever for two months and painful swelling of the right proximal leg for 10 days. Fever was continuous, with occasional chills and rigor, and a maximum recorded temperature of 104-degree Fahrenheit. He had no history of headache, chest pain, cough, abdominal pain, jaundice, or dysuria. Ten days back, he suddenly started experiencing continuous nonradiating throbbing pain in the right proximal leg accompanied by swelling. There was no history of trauma. He did not have a history of diabetes mellitus or hypertension.

He traveled to Malaysia as a migrant worker where he stayed for four years from 2002 to 2006. After he returned from Malaysia, he stayed in his home, in eastern Nepal doing farming. Later in 2018, he decided to leave for Goa, India to find a job. He was asymptomatic in between and had no similar complaint in the past. It had been four months since he started working as a security guard in Goa when he started developing the above-mentioned symptoms and returned to Nepal for treatment. He was then evaluated in a tertiary center where they suspected tuberculosis of the abdomen (Koch's abdomen) as the possible cause and started antitubercular drugs.

**Figure 1:**
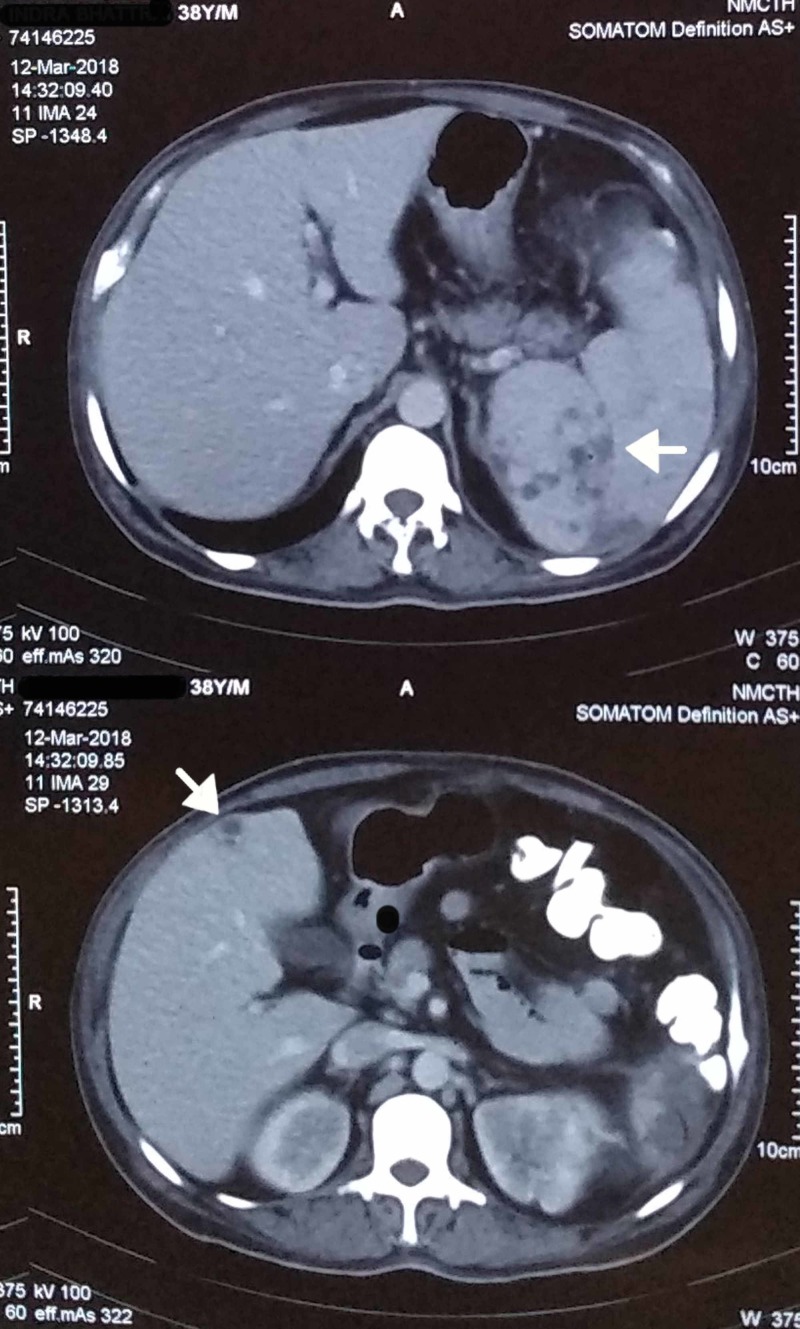
CECT of the abdomen showing hepatosplenomegaly with white arrows localizing ill-defined hypoechoic lesions in the liver and spleen, suggestive of abscesses. CECT, contrast-enhanced computed tomography

In the previous hospital, he was likely investigated under the impression of pyrexia of unknown origin as he presented with chief complaints of fever for more than a month, which was nonresolving and was associated with anorexia and weight loss. The patient never had any complaints of abdominal pain, constipation, or diarrhea. In order to rule out the cause of anorexia, contrast-enhanced computed tomography (CECT) of the abdomen, as well as a colonoscopy, were performed. CECT revealed hepatosplenomegaly with multiple ill-defined hypoechoic lesions suggestive of abscesses (Figure 1) and thickening of the ileocecal junction with adjacent lymphadenopathy. Colonoscopy biopsy suggested nonspecific colitis. These results might have led them to the differential of Koch’s abdomen.

On presentation to our institute, he was in a delirious state with a temperature of 103-degree Fahrenheit, a regular pulse rate of 120 beats per minute, sphygmomanometric blood pressure of 70/50 millimeters of mercury, respiratory rate of 30 per minute, and oxygen saturation of 97% at ambient air. He was pale, icteric, and had tender hepatomegaly. Other systemic findings were normal. Right proximal leg revealed a shiny, erythematous, tender swelling with diffuse edema extending to the knee joint. The local temperature was raised. The movement of the right knee joint was painfully restricted.

**Figure 2:**
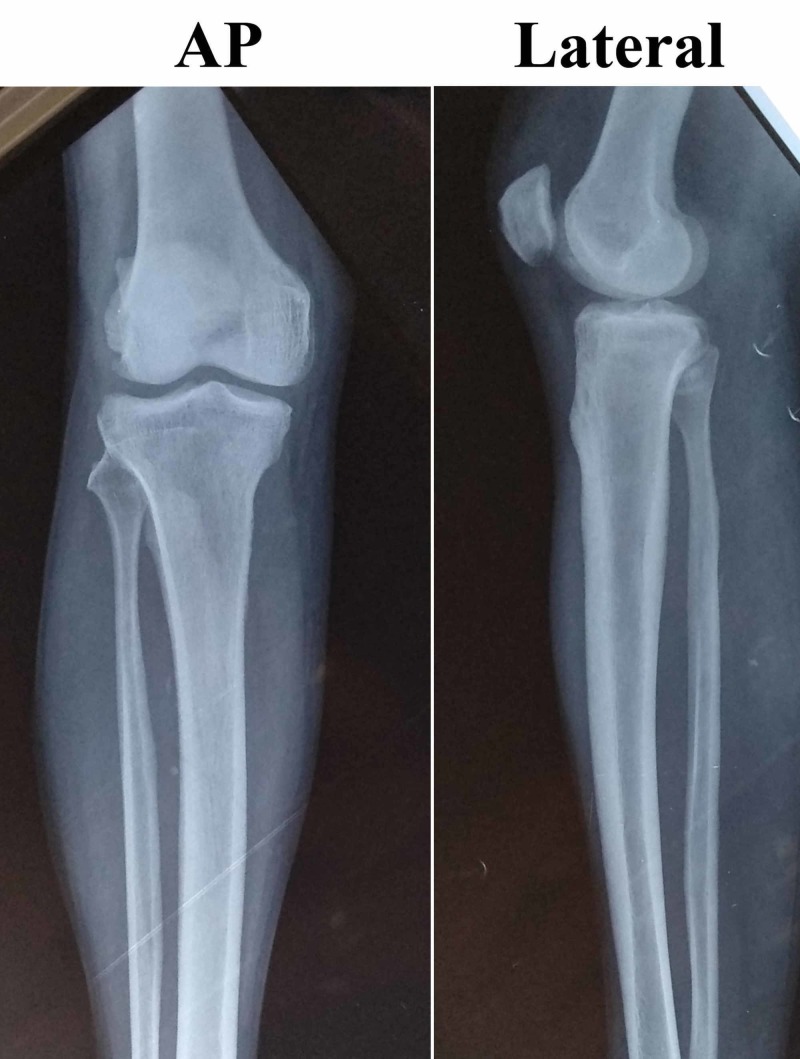
The preoperative X-ray of the right knee joint with proximal leg and distal thigh showing no osteomyelitic changes.

Investigations revealed the following: white blood cells count 9.02 × 109 /L (normal range: 4-11 × 109), platelets 6.5 × 1010 /L (normal range: 1.5-4.5 × 1011), hemoglobin 80 g/L (normal range: 115-160), total bilirubin 66 µmol/L (normal range: 3-21), direct bilirubin 34 µmol/L (normal value: <4), alanine aminotransferase 110 units/L (normal value: <42), aspartate aminotransferase 104 units/L (normal value: <37), alkaline phosphatase 300 units/L (normal range: 38-94), urea 12.7 mmol/L (normal range: 1.6-7), creatinine 176 mmol/L (normal range for male: 60-110), sodium 123 mEq/ L (normal range: 135-146), potassium 2.9 mEq/ L (normal range: 3.5-5.2), random blood sugar of 6 mmol/ L (normal range: 3.5-7), erythrocytic sedimentation rate of 75 mm/hour (normal range for male: 0-15 mm/hour), negative C-reactive protein and negative spot tests for human immune-deficiency virus antibody, hepatitis B virus surface antigen and hepatitis C virus antibody. X-ray of the right leg was normal (Figure 2).

 

**Figure 3:**
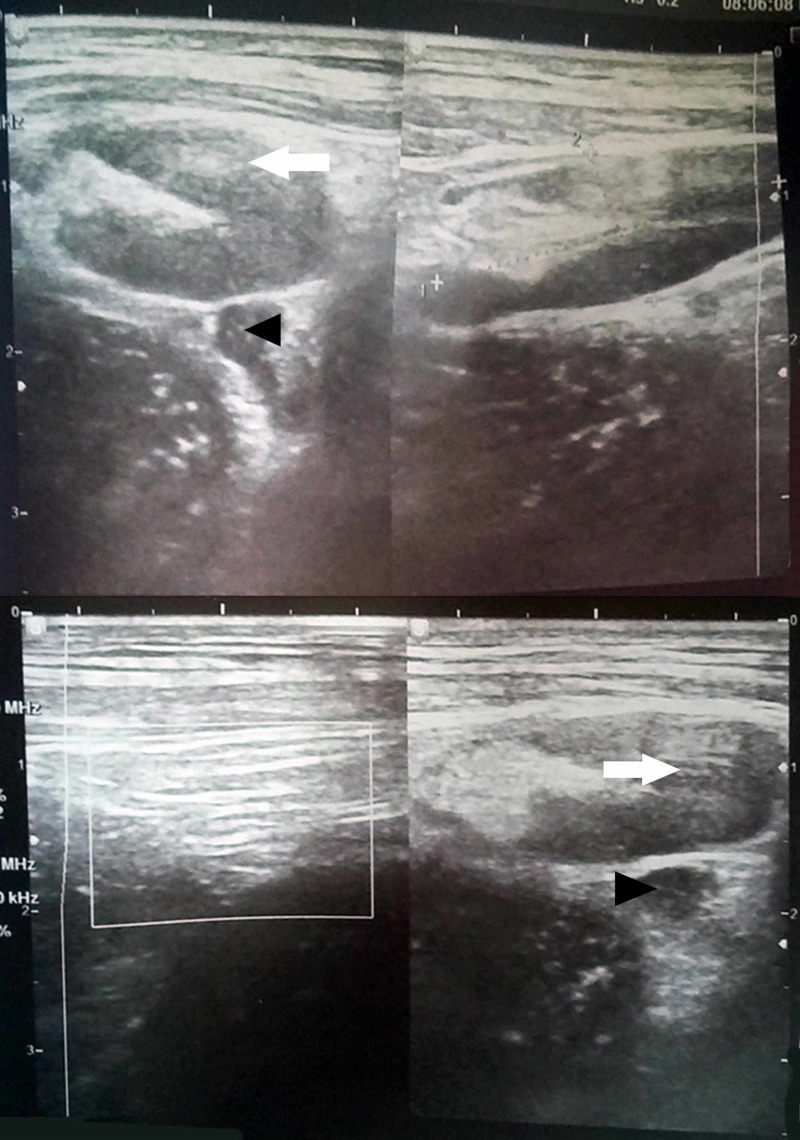
USG showing soft tissue collection (white arrows) and sub-periosteal collection (black arrowheads) over anteriomedial aspect of the right proximal leg suggestive of acute osteomyelitis. USG, ultrasonography

Ultrasonography (USG) was suggestive of acute osteomyelitis of the right proximal tibia (Figure 3).

MRI was not done before drainage, as we were pretty confident in our clinical diagnosis, and USG too had identified subperiosteal collection. In addition to these reasons, we were also limited by the poor financial status of our patient. Blood culture samples showed no growth.

**Figure 4:**
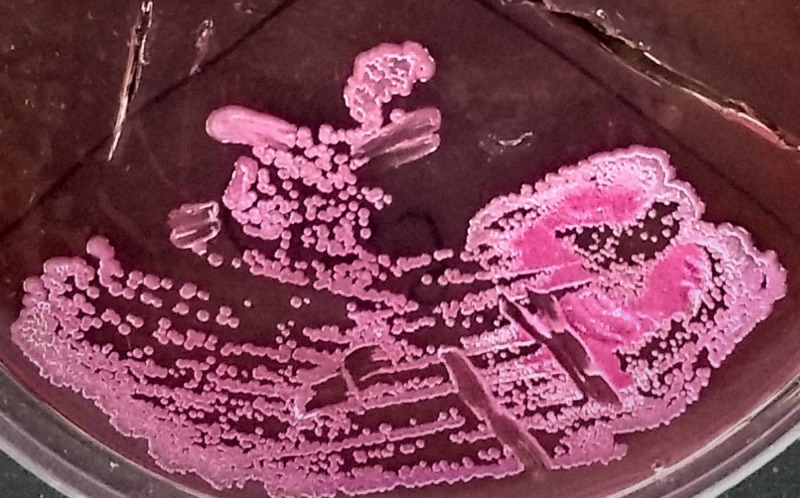
Culture on MacConkey agar showing wrinkled pink colony after 48 h of incubation.

As there was swelling around the right knee joint, knee aspiration was done and seropurulent viscous fluid was aspirated. Suspecting to be septic arthritis of the knee joint, arthrotomy was done and the synovial sample was taken. Approximately 10 mL of blood mixed pus was drained from the anteromedial aspect of the proximal tibia. The cortex of proximal tibia was drilled through which frank pus came, and then the cortical window was made. The pus and the synovial fluid were sent for Gram's stain, culture, and sensitivity. A synovial biopsy was sent for histopathological examination that reported negative for malignancy. Gram's stain of pus and synovial fluid revealed Gram-negative, safety pin-shaped bacteria suggestive of *B. pseudomallei*. The same was confirmed by the culture (Figure 4) which we considered being the gold standard for confirming our diagnosis.

After obtaining a culture report, we started ceftazidime (two grams, intravenously, every eight hourly) along with levofloxacin (750 milligrams, intravenously, once daily) as initial intensive therapy for six weeks. The patient was gradually mobilized and discharged on oral eradication therapy with cotrimoxazole (one double-strength tablet twice daily) and doxycycline (100 milligrams twice daily) with supplemented folic acid. Eradication therapy was continued for six months. No features suggestive of adverse drug reactions were reported during the entire duration of treatment.

**Figure 5:**
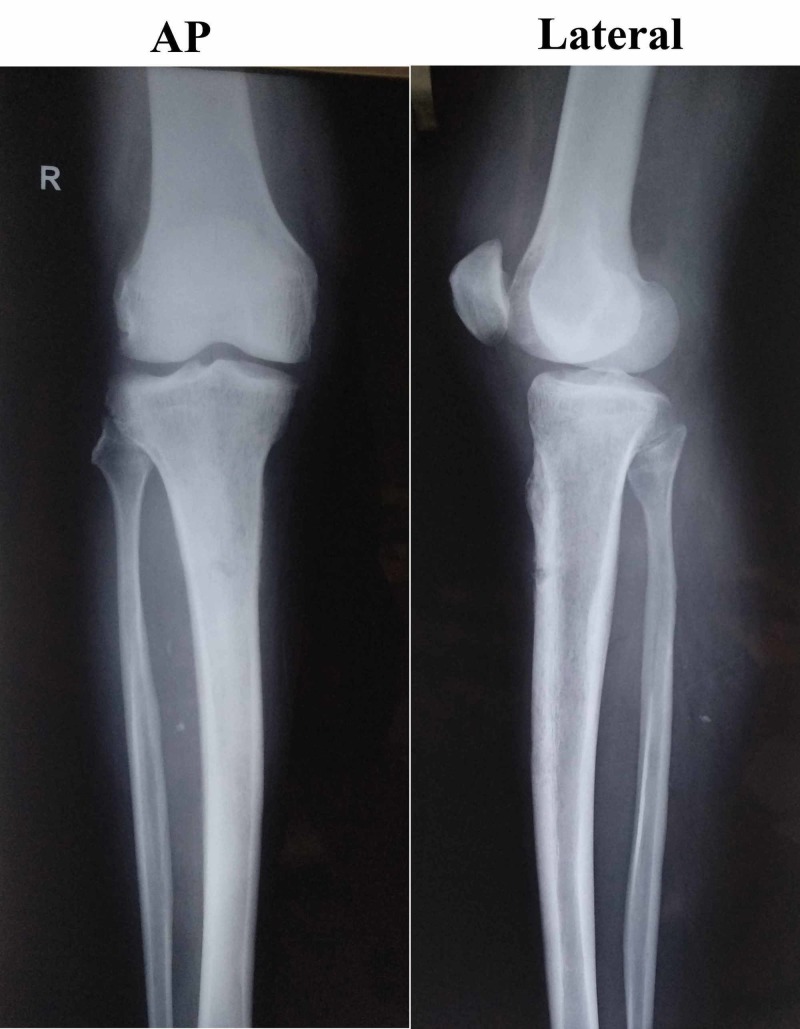
A one-month post-operative X-ray of the right knee joint with proximal leg and distal thigh, still not showing features of osteomyelitic changes.

X-ray of the right leg done just before the discharge was normal (Figure 5) and repeat USG of abdomen and pelvis showed resolution of hepatic and splenic abscesses.

**Figure 6:**
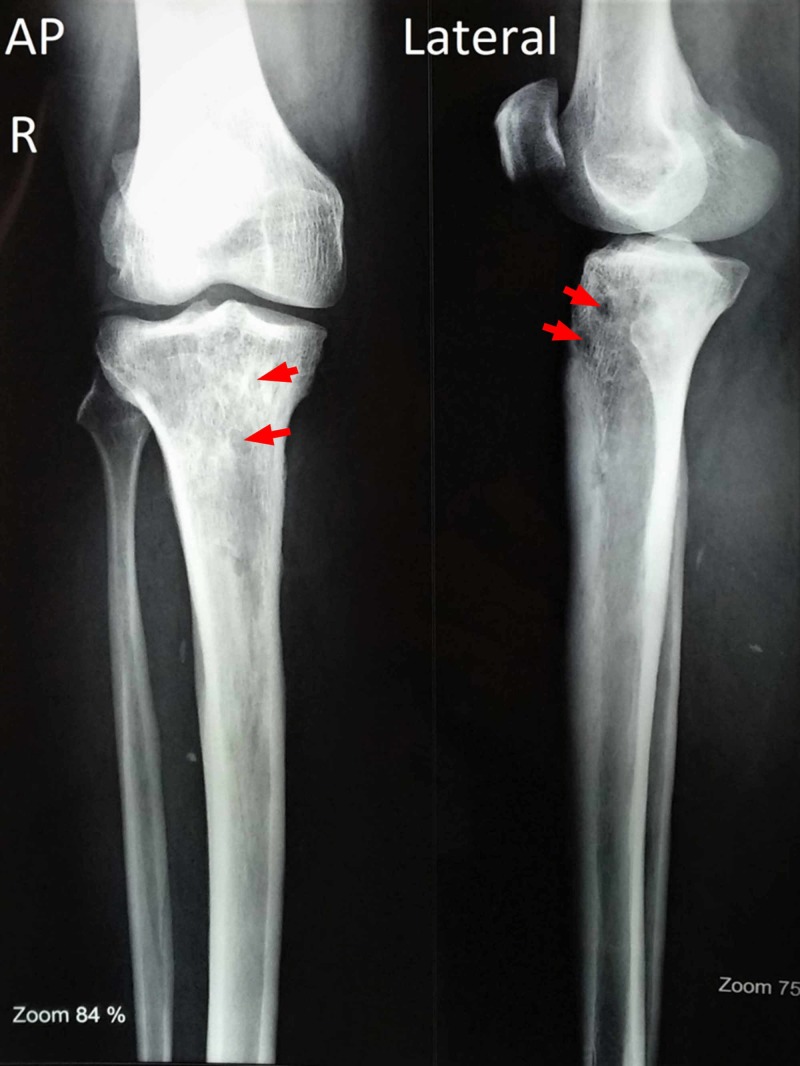
X-ray of right leg taken six months postoperatively showing few lytic lesions (red arrows) in the proximal tibia.

He was regularly followed up until six months and was fully compliant with medications. No features suggestive of adverse drug reactions were reported during the entire duration of treatment. X-ray of the right leg done during his last visit revealed few lytic areas in the proximal tibia (Figure 6).

However, he had no pain or tenderness in the right proximal leg with a normal range of motion of the knee joint and was bearing the full amount of body weight. He was counseled for a twice-yearly follow-up.

## Discussion

The known distribution of melioidosis is referred to as "tip of the iceberg" as the cases are infrequently reported [5]. Moreover, the cases from the Indian subcontinent (India, Pakistan, Bangladesh, Nepal, Sri Lanka, Maldives) are underreported although the case volume is expected to be high [6]. The absence of a surveillance system poses a major challenge to determine the magnitude of this problem. After the first case was reported in Nepal in 2004 [7], two more cases have been reported after a gap of about 15 years [8]. Interestingly, those patients had similarly traveled to Malaysia but developed pneumonia which is the most common presentation [3]. As many Nepalese migrant workers travel to countries endemic to melioidosis like Thailand [9] and Malaysia, they might 'import' the disease along with them. In addition to this, melioidosis is often confused with more common infectious diseases like tuberculosis [4]. Similarly, the lack of awareness among the clinicians cannot be overlooked in this regard.

In our case, we are not quite sure whether he acquired the infection in Malaysia or South India. Normally, the incubation period ranges from 1 to 21 days [10]. In the former case, the infection might have remained latent until presentation, as *B. pseudomallei* can remain latent until subsequent reactivation for as long as 62 years [11].

Skin inoculation is the most common route infection, followed by inhalation or ingestion [1]. Some 75%-81% of cases occur during the monsoonal wet season [1]. Unless the risk factors are present, a healthy person is unlikely to be affected by melioidosis. Diabetes is the most important risk factor for melioidosis followed by heavy alcohol use, chronic pulmonary disease, chronic renal disease, etc. [3, 12-14]. To our surprise, none of the above risk factors were present in our patient. The low platelet counts noted initially might be due to sepsis which fortunately improved upon subsequent treatment.

Pneumonia is the most common presenting feature, followed by genitourinary infection, skin, and soft tissue infection [3]. Abscess of the brain and visceral organs like spleen and liver are less common [3]. Bone and joint infections are rare features [12] making them difficult to differentiate from other causes of infection like tuberculosis [4] except that systemic features become more prominent [15]. Misdiagnosis as tuberculosis often leads to mistreatment which is very well exemplified in our case. The antitubercular drugs cause a wide array of adverse effects, often feared drug-induced hepatitis. However, some coexisting features are often helpful in the diagnosis. In a patient with osteomyelitis, coexisting involvement of the lung and visceral organs favors the diagnosis of melioidosis [16]. Septic arthritis is the most common manifestation of musculoskeletal melioidosis, followed by osteomyelitis, pyomyositis, and soft tissue abscess [14].

MacConkey agar and blood agar are routinely used for its culture. Gram's stain and histopathological stains are not specific for the organism, hence they do not aid in the diagnosis [1]. Routine imaging is necessary due to the common occurrence of varied internal abscesses [17].

Ceftazidime is the treatment of choice in the acute intensive phase [18]. The intensive therapy should be continued for four weeks in the case of septic arthritis [18]. After the initial intensive therapy has been provided, the consequent eradication therapy has been considered essential to prevent recrudescence or later relapses of melioidosis [18]. Cotrimoxazole should normally be considered the initial eradication agent of choice for melioidosis globally [18]. The duration of eradication therapy should be three to six months [19]. Liver, spleen, and renal abscesses respond to prolonged antibiotic therapy [18], while prostatic abscesses usually require drainage in addition to antibiotics [20]. Septic arthritis usually requires operative drainage and washouts [18]. Though patients with rheumatological involvement have a higher chance of recurrence [14] our patient was doing well till the last follow-up.

## Conclusions

Due to the wide spectrum of presentation and a long latency period, the diagnosis of melioidosis is challenging to many physicians in nonendemic countries. Diagnosis of melioidosis requires a high degree of suspicion among clinicians and microbiologists, and an elaborate travel history to endemic areas. Severe melioidosis may occur even in the absence of established risk factors. Early diagnosis would prevent fatal consequences. Maybe the time has come to initiate an effective surveillance system that would provide more information about the clinical characteristics and distribution of this disease within nonendemic countries like Nepal.
